# FedSCOPE: Federated cross-domain sequential recommendation with decoupled contrastive learning and privacy-preserving semantic enhancement

**DOI:** 10.1038/s41598-026-38628-y

**Published:** 2026-02-05

**Authors:** Lujin Zhao, Yi Lin, Sujuan Qin, Wenmin Li, Fei Gao, Yijie Shi, Zhengping Jin

**Affiliations:** 1 School of Cyberspace Security, Beijing University of Posts and Telecommunications, Beijing, 100876 China; 2 State Key Laboratory of Networking and Switching Technology, Beijing University of Posts and Telecommunications, Beijing, 100876 China

**Keywords:** Federated Learning, Cross-Domain Sequential Recommendation, Contrastive Learning, Large Language Models, Computational biology and bioinformatics, Mathematics and computing

## Abstract

Cross-domain sequential recommendation (CDSR) models users’ dynamic preferences by exploiting behavioral signals from multiple domains, but it faces challenges in data sparsity, domain heterogeneity, and privacy protection. Although federated learning enables privacy-preserving CDSR by keeping raw data local, existing methods often suffer from sparse representations, unstable cross-domain alignment, and severe utility degradation under uniform differential privacy. In this work, we propose FedSCOPE, a novel federated CDSR framework that addresses these challenges through three tightly coupled and explicitly aligned components. First, FedSCOPE enriches user and item representations via offline large language model (LLM)-generated semantic augmentation, mitigating sparsity while avoiding online LLM inference and the associated privacy and deployment risks. Second, it introduces an Intra- and Inter-Domain Decoupled Contrastive Learning mechanism that separates intra-domain personalization from inter-domain discrimination, enabling robust cross-domain alignment under heterogeneous data distributions. Third, FedSCOPE incorporates an adaptive personalized differential privacy strategy that dynamically allocates privacy budgets and clipping thresholds according to client-specific data characteristics, achieving a more favorable privacy–utility trade-off in federated environments. These components are jointly optimized within a secure federated learning framework. Extensive experiments on multiple real-world datasets demonstrate that FedSCOPE consistently outperforms state-of-the-art baselines, achieving higher recommendation accuracy, stronger cross-domain generalization, and improved privacy–utility balance.

## Introduction

Sequential recommendation (SR)^1^ has become a fundamental technique in modern personalization systems by modeling user–item interaction sequences to predict future user preferences. However, as users increasingly engage with multiple online services across different platforms, single-domain recommendation models^2,3^ struggle to capture users’ evolving interests across diverse contexts. To address this limitation, cross-domain sequential recommendation (CDSR)^4,5^ integrates user behaviors from multiple domains, enabling richer user representations and improved recommendation performance. CDSR has shown strong potential in real-world applications such as e-commerce, media platforms, and online content services.

The growing interconnection of digital ecosystems further amplifies the importance of CDSR. User behaviors are no longer isolated within a single application but instead span multiple services, forming complex and interdependent preference patterns. For example, interactions on a book-reading platform may reveal latent interests that are highly informative for video streaming or shopping recommendations. Effectively leveraging such cross-domain behavioral signals is crucial for building context-aware and proactive recommender systems. Without appropriate mechanisms to model these interactions, recommendation systems remain siloed and fail to fully exploit users’ behavioral histories.

Despite its effectiveness, most existing CDSR approaches rely on centralized data aggregation, which introduces serious privacy risks and raises compliance concerns under data protection regulations such as GDPR. To overcome these issues, federated cross-domain sequential recommendation (FedCDSR)^6,7^ has emerged as a promising paradigm. FedCDSR enables multiple platforms or clients to collaboratively learn cross-domain knowledge while keeping raw user data local, thereby providing a privacy-preserving solution for cross-domain personalization.

However, current FedCDSR methods still face three fundamental challenges. First, data sparsity is particularly severe in niche or long-tail domains, where limited interaction histories make it difficult to learn expressive user and item representations, leading to degraded recommendation accuracy and cold-start issues. Second, domain heterogeneity caused by imbalanced data distributions across clients often results in inconsistent local model updates, which slows global convergence and may even induce negative knowledge transfer across domains. Third, the trade-off between privacy protection and model utility remains challenging: conventional differential privacy (DP) mechanisms typically adopt uniform noise injection across all clients, which is poorly suited to heterogeneous federated environments and often causes unnecessary utility degradation.

Although several representative methods attempt to address subsets of these challenges, they still suffer from notable limitations. For example, FELLAS^8^ introduces large language models (LLMs) to enrich semantic representations in federated sequential recommendation. However, its reliance on remote LLM inference tightly couples the recommendation pipeline with external services, resulting in increased inference latency, additional computational and monetary costs, as well as potential exposure of user behavior data to third-party providers. These issues may significantly limit its deployability under strict privacy and compliance requirements. In contrast, FedCSR^9^ employs dual contrastive learning objectives to enhance cross-domain robustness but does not incorporate explicit semantic enrichment. Consequently, its performance can be severely limited in highly sparse or cold-start scenarios, where behavioral signals alone are insufficient to support expressive and stable representation learning.

These challenges indicate that an effective framework should simultaneously enrich sparse representations, align heterogeneous domain knowledge, and adapt privacy protection to client-specific characteristics. To jointly address these challenges, we propose FedSCOPE, a unified federated cross-domain sequential recommendation framework that explicitly aligns its core components with the three aforementioned challenges. To alleviate data sparsity, FedSCOPE incorporates an offline LLM-based semantic augmentation module that enriches user and item representations with high-level semantic attributes in a privacy-preserving manner, without relying on online LLM services during training or inference. To cope with domain heterogeneity, we design an Intra- and Inter-Domain Decoupled Contrastive Learning (IIDCL) mechanism, which disentangles intra-domain representation alignment from inter-domain uniformity, thereby improving personalization while preventing negative cross-domain interference. To address the privacy–utility trade-off, FedSCOPE adopts an adaptive personalized differential privacy mechanism that dynamically adjusts privacy budgets and clipping thresholds according to client-specific data characteristics, achieving a more favorable balance between privacy protection and model utility. These components are jointly optimized within a secure federated learning framework, enabling FedSCOPE to simultaneously enhance recommendation accuracy, robustness, and privacy.

The main contributions of this work are summarized as follows:

- We propose FedSCOPE, a federated cross-domain sequential recommendation framework that systematically addresses data sparsity, domain heterogeneity, and the trade-off between privacy and utility.
- We design three complementary components, namely an offline LLM-based semantic augmentation module, an Intra- and Inter-Domain Decoupled Contrastive Learning mechanism, and an adaptive personalized differential privacy strategy, and explicitly demonstrate how each component corresponds to a specific challenge in federated CDSR.
- Comprehensive experiments on multiple real-world datasets demonstrate that FedSCOPE consistently outperforms state-of-the-art methods, yielding improved recommendation accuracy, enhanced cross-domain generalization, and better privacy–utility trade-offs.

## Related work

### Cross-domain sequential recommendation

Cross-domain recommendation seeks to alleviate data sparsity by leveraging user–item interactions across multiple domains^10,11^. Early approaches such as NCF-MLP^12^ employed multilayer perceptrons to learn latent domain representations, while CoNet^13^ introduced cross-network connections for explicit knowledge transfer. However, these methods ignored temporal dynamics, limiting their effectiveness in sequential recommendation tasks. To overcome this, CDSR methods emerged: $\pi$-Net^14^ utilized gated recurrent units to capture evolving user preferences; PSJNet^15^ designed a split–merge architecture to jointly model multi-domain knowledge and user intent; LEA-GCN^16^ exploited graph neural networks to encode rich item–item relationships across domains. Beyond CDSR, recent progress in self-supervised and diffusion-enhanced recommendation has further strengthened representation learning. MSRec^17^ introduces a multi-view self-supervised framework on heterogeneous graphs, where meta-path based structural views and local–global contrastive learning capture complex semantics in graph data. KMDCL^18^ employs a mask diffusion mechanism to generate adaptive knowledge views and uses user-intent–guided denoising to enhance robustness in knowledge-aware recommendation. FACLK^19^ mitigates feature distortion in high-order knowledge propagation through a feature decorrelation constraint and adaptive semantic refinement, enabling more reliable contrastive learning. SDMMR^20^ integrates masked prediction with diffusion-based perturbation and a smoothing mechanism to alleviate multimodal noise and improve semantic alignment. These advances inspire our design of decoupled contrastive learning and semantic enhancement in FedSCOPE. However, all such methods assume centralized data access and cannot be directly applied in federated cross-domain scenarios, where privacy constraints fundamentally restrict representation sharing.

### Federated cross-domain recommendation

To overcome the limitations of centralized CDSR, federated learning (FL)^21^ has been explored as a privacy-preserving paradigm. Ammad et al.^22^ first introduced a federated collaborative filtering framework, inspiring subsequent works aimed at improving recommendation utility^23^ and enhancing privacy or security mechanisms^24,25^. Knowledge transfer under FL is particularly challenging due to non-IID data and restricted communication. FedCT^26^ employs variational autoencoders to exchange latent domain embeddings without exposing raw data; FedCTR^27^ builds a multi-platform training framework for CTR prediction; FedDCSR^7^ introduces decoupled representation learning to address feature heterogeneity and distribution shifts. More recent studies integrate advanced techniques. FELLAS^8^ incorporates large language models through online query services to enhance semantic transfer, but this approach raises additional privacy and operational concerns in FL settings. FedCSR^9^ applies contrastive learning to mitigate reliance on explicit cross-platform user alignment, yet its uniform differential privacy mechanism limits its effectiveness under heterogeneous client distributions. Compared with these methods, FedSCOPE adopts a different technical route by leveraging local offline LLM-based semantics to avoid exposure to external services, employing decoupled contrastive learning tailored for heterogeneous domains, and introducing an adaptive differential privacy mechanism that provides fine-grained protection. These distinctions further highlight that FedSCOPE is not a straightforward combination of existing centralized or federated techniques, but a federated-specific redesign that jointly considers semantic enrichment, domain heterogeneity, and privacy constraints. To better illustrate the distinctions, we provide a direct comparison of representative approaches in Table 1.

**Table 1:** Comparison of FedSCOPE with representative federated CDSR methods.

Method	Offline LLM	Decoupled CL	Adaptive DP
FELLAS^8^	$\times$ (Online)	$\times$ (Perturbation)	$\times$ (Uniform)
FedCSR^9^	$\times$ (None)	$\times$ (Dual)	$\times$ (Uniform)
**FedSCOPE (Ours)**	$\checkmark$	$\checkmark$	$\checkmark$

## Methodology

### Problem definition

**Fig. 1:**
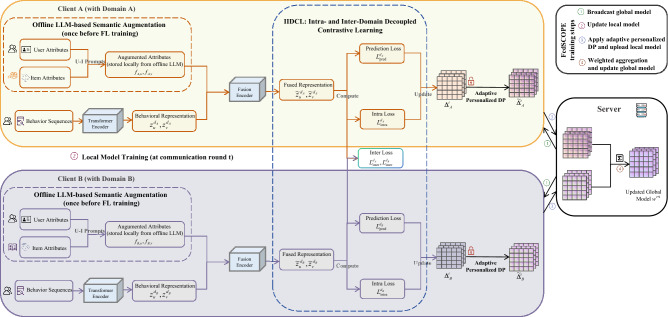
Overview of the proposed FedSCOPE framework.

We consider a FL system with *K* clients and a central server, spanning *m* domains $\mathcal {D}_{\text {total}}=\{d_1, \dots , d_m\}$. Each client may cover multiple domains and collaboratively trains a federated cross-domain sequential recommendation model with global parameters $\textbf{w}$. At communication round *t*, the server broadcasts the current global model parameters $\textbf{w}^t$ to a subset of selected clients. Each client performs local optimization on its private dataset $D_k$ and returns a privatized model update to the server for aggregation. For client *k*, the local dataset is denoted as $D_k=\{S_k^{(u)}\}_{u \in U_k}$, where $U_k$ is the set of users on client *k*. Each user behavior sequence is truncated or padded to the most recent *N* interactions: $S_k^{(u)}=(s_u^1, \dots , s_u^N)$. Each domain $d_i \in \mathcal {D}_{\text {total}}$ is associated with an item set $V_{d_i}$, and the unified item space is defined as $V^*=\bigcup _{i=1}^m V_{d_i}$. For each user, we maintain both domain-specific sequences $S_k^{d_i}$ and an aggregated cross-domain sequence $S_k^*$. Compared with single-domain sequential recommendation, FedCDSR faces three key challenges: (i) domain sparsity and imbalance, (ii) heterogeneous user behavior distributions that induce inconsistent client updates, and (iii) strict privacy constraints that limit direct information sharing. The learning objective is to predict the next interacted item at step $N{+}1$ either from a target domain $d_i$ or from the unified item space $V^*$, where all users and items are embedded into a shared *d*-dimensional latent space. To address these challenges, we propose FedSCOPE, a federated cross-domain sequential recommendation framework as illustrated in Fig. 1. Here, Domain A and Domain B in Fig. 1 are used as representative examples of domains $d_i \in \mathcal {D}_{\text {total}}$ to illustrate intra-domain modeling and inter-domain interaction, while FedSCOPE is generally applicable to an arbitrary number of domains.

### User-behavior sequence modeling and representation learning

To model user behavior sequences as the foundation for downstream semantic augmentation and contrastive objectives, we first encode historical interactions into latent representations using a Transformer-based encoder. This encoder captures long-range dependencies and enables efficient parallel computation, producing user representations $\textbf{z}_u^{d_i}$ that serve as the basis for cross-domain recommendation. For user *u*, the latest *N* interactions $S_k^{(u)}=(s_u^1,\dots ,s_u^N)$ are mapped to embeddings $\textbf{e}_{s_u^n}\in \mathbb {R}^d$, forming $E_k^{(u)}=(\textbf{e}_{s_u^1},\dots ,\textbf{e}_{s_u^N})$. To encode order, sinusoidal positions are added, i.e., $\textbf{e}_{s_u^n}^p=\textbf{e}_{s_u^n}+\textbf{p}_n$. These position-aware embeddings are processed by *L* Transformer layers with multi-head self-attention and feed-forward networks (with residuals, normalization, and dropout). The final representation in domain $d_i$ is:

1 $$\begin{aligned} \textbf{z}_u^{d_i} = \text {TransformerEnc}\!\left( \textbf{e}_{s_u^1}^p,\dots ,\textbf{e}_{s_u^N}^p\right) \in \mathbb {R}^d. \end{aligned}$$

We denote the Transformer-only output as $\textbf{z}$, the LLM-enhanced features as $f_A$, and the fused embedding as $\tilde{\textbf{z}}$. While $\textbf{z}_u^{d_i}$ captures intra-domain sequential patterns, it remains incomplete under sparsity and heterogeneity, motivating the LLM-based augmentation module.

### LLM-augmented user and item representations

Federated cross-domain data are often sparse and incomplete, especially under cold-start scenarios. To address this, we introduce an *offline* LLM-based augmentation module that enriches user profiles and item metadata via semantic reasoning (see Table 2).

**User-side augmentation.** For each client *k*, given a sequence $S_k^{(u)}$, prompts $P_u^U$ query the LLM to infer attributes beyond demographics and genre, such as social tendencies, viewing environments, consumption patterns, and cross-domain interests. The generated vectors $f_{A,u} \in \mathbb {R}^{d_{LLM}}$ are fused with sequential embeddings to form enriched user profiles.

**Item-side augmentation.** Many domains lack complete metadata. Given basic fields (title, category, year), prompts $P_v^I$ guide the LLM to infer extended attributes, e.g., directors, actors, production company, sub-genres, awards, and ratings. Unavailable fields (e.g., subjective ratings) are set to N/A. The resulting $f_{A,v} \in \mathbb {R}^{d_{LLM}}$ provides richer item representations, mitigating cold-start issues.

**Offline usage.** Unlike prior works relying on online LLM services, augmentation is done once before training. Clients sanitize prompts, query locally, and reuse generated attributes throughout training. Raw data never leaves the client, ensuring privacy-preserving enrichment without runtime dependency.

**Robustness to noisy LLM-generated attributes.** We acknowledge that LLM-generated attributes may contain noise or imperfect inferences. To mitigate their potential impact, we adopt several safeguards. First, all prompts enforce strict JSON schemas, and unavailable or uncertain attributes are explicitly marked as N/A, preventing the introduction of arbitrary or unstructured noise. Second, LLM-generated features are not used independently but are fused with behavior-driven representations through a lightweight MLP, allowing the model to learn appropriate weighting and downplay unreliable semantic signals. Third, the proposed IIDCL and federated training process further enhance robustness by emphasizing consistent behavioral patterns across users and domains, effectively regularizing noisy semantic features. As a result, occasional inaccuracies in LLM outputs do not dominate the learning process and have limited influence on final recommendations.

**Feature fusion.** Specifically, let $\textbf{z}_u^{d_i}$ and $\textbf{z}_v^{d_i}$ denote the behavior-driven sequential embeddings of user *u* and item *v* in domain $d_i$, and let $f_{A,u}$ and $f_{A,v}$ be their corresponding LLM-generated semantic features. To integrate behavioral and semantic information, we introduce a lightweight fusion encoder $\text {Enc}(\cdot )$, implemented as a two-layer multi-layer perceptron (MLP), which projects the concatenated features into a shared latent space. The encoder produces fused representations $\tilde{\textbf{z}}_u^{d_i}$ and $\tilde{\textbf{z}}_v^{d_i}$ as follows:

2 $$\begin{aligned} \tilde{\textbf{z}}_u^{d_i} = \text {Enc}([\textbf{z}_u^{d_i}; f_{A,u}]), \quad \tilde{\textbf{z}}_v^{d_i} = \text {Enc}([\textbf{z}_v^{d_i}; f_{A,v}]), \end{aligned}$$

where $\tilde{\textbf{z}}_u^{d_i}, \tilde{\textbf{z}}_v^{d_i} \in \mathbb {R}^d$. This fusion integrates sequential patterns with enriched semantics, improving accuracy, generalization, and robustness under cold-start scenarios.

**Table 2:** Prompt templates for offline LLM-based augmentation.

Task	Prompt Template
**User Profile**	System: You are a recommender data curator. User: Given the user’s movie history [title, year, genres], infer the following JSON fields: { ”age_range”: ””, ”gender”: ””, ”liked_genres”: [...], ”disliked_genres”: [...], ”social_tendencies”: ””, ”social_relationships”: ””, ”preferred_activities”: ””, ”viewing_environment”: ””, ”future_interests”: [...], ”language”: ”” } Rules: Follow schema; avoid PII; use concise and factual values.
**Item Attribute**	System: You complete missing metadata for movies. User: For the given movie [title, year, genres], output the following JSON fields: { ”director”: ””, ”country”: ””, ”language”: ””, ”main_actors”: [...], ”production_company”: ””, ”filming_locations”: [...], ”audience_ratings”: ”N/A” } Rules: Mark optional fields as ”N/A”; follow strict schema; provide short factual values.

### Intra- and inter-domain decoupled contrastive learning

To enhance intra-domain consistency and inter-domain discriminability, we propose Intra- and Inter-Domain Decoupled Contrastive Learning. Unlike the standard InfoNCE loss that entangles attraction and repulsion within a single log-softmax term, IIDCL explicitly decouples alignment and uniformity components, thereby enabling finer-grained control over positive and negative forces. This design is particularly advantageous in federated settings with heterogeneous data distributions and limited local samples. Compared with FedCSR^9^, which applies dual contrastive objectives across clients, IIDCL directly targets intra-domain coherence and inter-domain separability within a unified framework.

Specifically, IIDCL consists of two complementary objectives. Intra-domain contrastive learning encourages an anchor user to attract a set of Top-*K* behaviorally similar users while repelling dissimilar ones, reinforcing preference consistency within each domain. Inter-domain contrastive learning aligns representations of the same user across different domains while pushing apart representations of different users, ensuring cross-domain separability. This decoupled design preserves personalized signals at the domain level while facilitating effective cross-domain knowledge transfer.

Formally, let $\mathcal {U}_{d_i}$ denote the user set of domain $d_i$. For a user $u \in \mathcal {U}_{d_i}$, we denote by $\textbf{z}_u^{d_i} \in \mathbb {R}^d$ the behavior-driven sequence representation obtained from the Transformer encoder, and by $\tilde{\textbf{z}}_u^{d_i} \in \mathbb {R}^d$ the fused representation after LLM-based semantic augmentation. Cosine similarity between two users $u_a$ and $u_b$ in domain $d_i$ is defined as $\text {sim}(u_a,u_b) = \frac{\tilde{\textbf{z}}_{u_a}^{d_i} \cdot \tilde{\textbf{z}}_{u_b}^{d_i}}{\Vert \tilde{\textbf{z}}_{u_a}^{d_i}\Vert _2 \, \Vert \tilde{\textbf{z}}_{u_b}^{d_i}\Vert _2}$.

For each user *u*, the Top-*K* most similar users are treated as positive samples, and all remaining users are regarded as negatives:

3 $$\begin{aligned} \mathcal {P}_u^{d_i} = \operatorname {TopK}_{u' \ne u}\big (\text {sim}(u,u'), K\big ), \quad \mathcal {N}_u^{d_i} = \mathcal {U}_{d_i} \setminus \big (\mathcal {P}_u^{d_i} \cup \{u\}\big ). \end{aligned}$$

The hyperparameter *K* controls the locality of the intra-domain neighborhood. A small *K* enforces strong local consistency but may be unstable under sparse data, while a large *K* risks introducing noisy positives. Following prior practice, we set $K = \min (K_{\max }, \lfloor \rho \cdot |\mathcal {U}_{d_i}| \rfloor )$ with $\rho = 0.02$ and $K_{\max } = 50$, and observe stable performance under moderate variations.

The intra-domain loss is defined as

4 $$\begin{aligned} \begin{aligned} \mathcal {L}_{\text {intra}}^{d_i} = \tfrac{1}{|\mathcal {U}_{d_i}|} \sum _{u \in \mathcal {U}_{d_i}} \Big [&-\lambda ^{a}_{\text {intra}} \tfrac{1}{|\mathcal {P}_u^{d_i}|} \sum _{u^+\in \mathcal {P}_u^{d_i}} \tfrac{\text {sim}(\tilde{\textbf{z}}_u^{d_i},\tilde{\textbf{z}}_{u^+}^{d_i})}{\tau _{\text {intra}}} + \lambda ^{u}_{\text {intra}} \log \!\Big (\sum _{u^-\in \mathcal {N}_u^{d_i}} \exp (\tfrac{\text {sim}(\tilde{\textbf{z}}_u^{d_i},\tilde{\textbf{z}}_{u^-}^{d_i})}{\tau _{\text {intra}}})\Big ) \Big ], \end{aligned} \end{aligned}$$

where $\mathcal {P}_u^{d_i}$ and $\mathcal {N}_u^{d_i}$ denote the positive and negative user sets for anchor user *u* in domain $d_i$, respectively. $\lambda ^{a}_{\text {intra}}$ and $\lambda ^{u}_{\text {intra}}$ control the strengths of the alignment and uniformity terms, and $\tau _{\text {intra}}$ is the temperature parameter. Although $\mathcal {N}_u^{d_i}$ is defined as the set of all non-positive users for notational clarity, in practice we randomly sample a fixed-size subset $\tilde{\mathcal {N}}_u^{d_i} \subset \mathcal {N}_u^{d_i}$ with $|\tilde{\mathcal {N}}_u^{d_i}| = M$ to construct the intra-domain loss. This negative sampling strategy significantly reduces computational cost while preserving sufficient diversity among negative examples. Unless otherwise stated, we set $M=100$ in all experiments.

For inter-domain contrastive learning, positive samples correspond to fused representations of the same user observed in different domains, while negative samples are fused representations of other users from those domains:

5 $$\begin{aligned} \mathcal {P}_u^{\text {inter}, d_i} = \{ \tilde{\textbf{z}}_u^{d_j} \mid d_j \ne d_i \}, \quad \mathcal {N}_u^{\text {inter}, d_i} = \{ \tilde{\textbf{z}}_{u'}^{d_j} \mid d_j \ne d_i,\, u' \ne u \}. \end{aligned}$$

The inter-domain contrastive objective is given by

6 $$\begin{aligned} \begin{aligned} \mathcal {L}_{\text {inter}}^{d_i} = \frac{1}{|\mathcal {U}_{d_i}|} \sum _{u \in \mathcal {U}_{d_i}} \Bigg [&-\lambda ^{a}_{\text {inter}} \frac{1}{|\mathcal {P}_u^{\text {inter}, d_i}|} \sum _{p \in \mathcal {P}_u^{\text {inter}, d_i}} \frac{\text {sim}(\tilde{\textbf{z}}_u^{d_i}, p)}{\tau _{\text {inter}}} + \lambda ^{u}_{\text {inter}} \log \!\Bigg ( \sum _{n \in \mathcal {N}_u^{\text {inter}, d_i}} \exp \!\left( \frac{\text {sim}(\tilde{\textbf{z}}_u^{d_i}, n)}{\tau _{\text {inter}}} \right) \Bigg ) \Bigg ], \end{aligned} \end{aligned}$$

where $\lambda ^{a}_{\text {inter}}$ and $\lambda ^{u}_{\text {inter}}$ balance alignment and uniformity, and $\tau _{\text {inter}}$ controls inter-domain smoothness.

The overall IIDCL objective is defined as

7 $$\begin{aligned} \mathcal {L}_{\text {IIDCL}} = \sum _{d_i} \Big ( \lambda _{\text {intra}} \mathcal {L}_{\text {intra}}^{d_i} + \lambda _{\text {inter}} \mathcal {L}_{\text {inter}}^{d_i} \Big ). \end{aligned}$$

Privacy is preserved since all intra-domain contrastive signals are computed locally on each client without any cross-domain data exchange. For inter-domain representation alignment, FedSCOPE adopts a Private Set Intersection (PSI) protocol^28^, which enables the identification of anonymized user overlaps across domains while ensuring that no raw user identifiers are disclosed. Importantly, FedSCOPE does not assume globally shared or centralized user identities. When multiple domains reside on the same platform or share consistent pseudonymous user identifiers, inter-domain alignment can be performed directly. For cross-platform scenarios without explicit identifier sharing, privacy-preserving user matching mechanisms (e.g., PSI-based secure linkage) are employed to identify a partial set of overlapping users without revealing raw identities. Inter-domain user alignment is an optional component rather than a strict prerequisite of FedSCOPE. When no reliable inter-domain alignment is available, the inter-domain contrastive objective is disabled, and the training process naturally degrades to pure intra-domain learning. In this case, the framework remains fully functional and privacy-preserving, while the strength of cross-domain knowledge transfer is reduced accordingly.

### Multi-task joint optimization and secure aggregation

For user $u \in \mathcal {U}_{d_i}$, the Transformer encoder produces a behavior-driven sequence representation $\textbf{z}_u^{d_i}\in \mathbb {R}^d$, which is further enriched by the offline LLM-based semantic augmentation module into $\tilde{\textbf{z}}_u^{d_i}$. Items in domain $d_i$ are represented analogously as $\tilde{\textbf{z}}_v^{d_i}$. Let $\mathcal {V}_{d_i}$ denote the candidate item set in domain $d_i$. Given $\tilde{\textbf{z}}_u^{d_i}$ and $\tilde{\textbf{z}}_v^{d_i}$, prediction scores $\hat{y}_{u,v}^{d_i} = \tilde{\textbf{z}}_u^{d_i} \cdot \tilde{\textbf{z}}_v^{d_i}$ are computed via inner product, and optimized using a binary cross-entropy objective. Formally, for each domain $d_i$, the next-item prediction loss is defined as

8 $$\begin{aligned} \mathcal {L}_{\text {pred}}^{d_i} = - \sum _{u \in \mathcal {U}_{d_i}} \sum _{v \in \mathcal {V}_{d_i}} \Big [ y_{u,v}^{d_i} \log \sigma (\hat{y}_{u,v}^{d_i}) + (1 - y_{u,v}^{d_i}) \log \big (1 - \sigma (\hat{y}_{u,v}^{d_i})\big ) \Big ], \end{aligned}$$

where $y_{u,v}^{d_i}\in \{0,1\}$ denotes the ground-truth interaction label and $\sigma (\cdot )$ is the sigmoid function. In practice, negative sampling is applied to reduce computational cost.

To jointly exploit behavioral, semantic, and structural signals, we combine the prediction objective with intra- and inter-domain decoupled contrastive learning. The overall local training objective for each client is

9 $$\begin{aligned} \mathcal {L} = \sum _{d_i} \Big ( \mathcal {L}_{\text {pred}}^{d_i} + \alpha \big ( \lambda _{\text {intra}} \mathcal {L}_{\text {intra}}^{d_i} + \lambda _{\text {inter}} \mathcal {L}_{\text {inter}}^{d_i} \big ) \Big ), \end{aligned}$$

where $\alpha$ balances the prediction loss and contrastive objectives, and $\lambda _{\text {intra}}$ and $\lambda _{\text {inter}}$ control the relative contributions of intra- and inter-domain contrastive learning, respectively. This joint objective yields three complementary effects: (i) $\mathcal {L}_{\text {pred}}^{d_i}$ drives accurate next-item recommendation; (ii) $\mathcal {L}_{\text {intra}}^{d_i}$ enhances domain-level personalization by encouraging consistency among behaviorally similar users; and (iii) $\mathcal {L}_{\text {inter}}^{d_i}$ aligns user representations across domains while preserving inter-user discrimination, thereby improving cross-domain generalization.

### Privacy protection

While the multi-task objective jointly optimizes semantic, behavioral, and structural signals, federated training must also satisfy strict privacy constraints. To this end, we incorporate an adaptive personalized differential privacy mechanism into the federated optimization pipeline. The proposed mechanism is applied at the client side in each communication round, after local optimization and before transmitting updates to the server. Combined with secure aggregation, this design ensures that all communicated updates satisfy client-level DP while preventing the server from accessing raw data or exact gradients. This design choice provides a practical balance between strong privacy guarantees and learning utility, making it suitable for real-world federated recommendation deployments. In FedSCOPE, differential privacy is enforced at the client-update level. Each communicated update corresponds to an aggregation over all users on a client, and the applied DP mechanism provides formal client-level privacy guarantees, ensuring that the contribution of any individual user’s behavior cannot be inferred from the transmitted updates.

Formally, a randomized mechanism $\mathcal {M}$ satisfies $(\epsilon ,\delta )$-DP if, for any pair of neighboring datasets *D* and $D'$, it holds that $\Pr [\mathcal {M}(D)\!\in \!\mathcal {S}] \le e^\epsilon \Pr [\mathcal {M}(D')\!\in \!\mathcal {S}] + \delta$ for all measurable sets $\mathcal {S}$. Smaller values of $\epsilon$ correspond to stronger privacy guarantees.

**Clipping and noise.** After minimizing the local objective at communication round *t*, each client *k* clips its update $\Delta _k^{t}$ with threshold $C_k^{t}$ and injects Gaussian noise. Specifically, the clipped update is computed as

10 $$\begin{aligned} \Delta _k^{t,\text {clip}} = \Delta _k^{t} \cdot \min \!\Big (1,\tfrac{C_k^{t}}{\Vert \Delta _k^{t}\Vert _2}\Big ), \end{aligned}$$

and the privatized update is obtained by adding Gaussian noise,

11 $$\begin{aligned} \tilde{\Delta }_k^{t} = \Delta _k^{t,\text {clip}} + \mathcal {N}(0,(\sigma _k^{t})^2 I), \end{aligned}$$

where the noise scale $\sigma _k^{t}$ is determined by the assigned privacy budget $(\epsilon _k,\delta _k)$ and satisfies

12 $$\begin{aligned} \sigma _k^{t} \ge \tfrac{\sqrt{2\ln (1.25/\delta _k)} \, C_k^{t}}{\epsilon _k}. \end{aligned}$$

**Adaptive allocation.** To account for client heterogeneity, privacy budgets are allocated proportionally to data size following $\epsilon _k = \epsilon _{\text {total}} \frac{|U_k|^{\beta }}{\sum _j |U_j|^{\beta }}$ and $\delta _k = \delta _{\text {total}}/K$, where $\beta \in [0,1]$ controls the degree of personalization. When $\beta =0$, the mechanism reduces to uniform DP, while larger $\beta$ assigns relatively larger budgets to data-rich clients. Unless otherwise stated, we use $\beta =0.5$, and observe stable behavior for $\beta \in [0.3,0.7]$.

To further reduce excessive utility loss, clipping thresholds are adapted to client-specific update magnitudes according to

13 $$\begin{aligned} C_k \leftarrow \gamma \cdot \operatorname {median}\!\left( \{\Vert \Delta _k^{(t')}\Vert _2\}_{t'=t-M}^{t-1}\right) , \end{aligned}$$

where *M* denotes the window size and $\gamma$ is a scaling factor.

**Privacy accounting.** The cumulative privacy loss across *T* communication rounds is tracked using a Rényi/moments accountant under the subsampled Gaussian mechanism. Given the noise multiplier $\mu _k=\sigma _k/C_k$ and sampling rate $q_k$, the accountant computes the accumulated guarantee $(\epsilon _k^{(T)},\delta _k)$. If a client approaches its privacy budget limit, the noise scale is increased or the client is temporarily suspended.

Privacy protection in FedSCOPE is enforced entirely at the client side and seamlessly integrated into the federated optimization process, providing strong client-level guarantees without compromising cross-domain collaborative learning.

### Federated cross-domain recommendation model training

The federated training procedure integrates the above privacy mechanism in a plug-and-play manner. Specifically, local optimization, privacy sanitization, and secure aggregation are sequentially performed within each communication round. In each communication round *t*, the server broadcasts the current global model parameters $\textbf{w}^t$ to a subset of selected clients. Each selected client performs local optimization by minimizing the joint objective $\mathcal {L}$ defined above, using only its private data.

After local training at communication round *t*, each client *k* computes a model update

14 $$\begin{aligned} \Delta _k^{t} = \textbf{w}_k^{t+1} - \textbf{w}^{t}, \end{aligned}$$

where $\textbf{w}_k^{t+1}$ denotes the locally updated model on client *k*. To satisfy client-level privacy guarantees, the update $\Delta _k^{t}$ is clipped and perturbed by the proposed adaptive personalized differential privacy mechanism, resulting in a privatized update $\tilde{\Delta }_k^{t}$. The server aggregates the privatized client updates via secure aggregation and updates the global model as

15 $$\begin{aligned} \textbf{w}^{t+1} = \textbf{w}^{t} + \frac{1}{\sum _{k=1}^K |U_k|} \sum _{k=1}^{K} |U_k| \cdot \tilde{\Delta }_k^{t}, \end{aligned}$$

where $|U_k|$ denotes the number of users on client *k*. Secure aggregation ensures that the server cannot access any individual client update, while the injected noise provides formal client-level $(\epsilon _k,\delta _k)$ differential privacy guarantees. This federated optimization procedure enables FedSCOPE to collaboratively learn a global cross-domain recommendation model with formal privacy guarantees, while preserving robustness across multiple domains.

## Experiments

To comprehensively evaluate the effectiveness and practicality of the proposed FedSCOPE framework, we design a series of experiments aimed at answering the following key research questions (RQs):

- **RQ1:** Does FedSCOPE consistently improve the performance of federated cross-domain sequential recommendation compared with representative baselines?
- **RQ2:** How much do LLM-based semantic enhancements of user and item representations contribute to the overall performance, and how sensitive is the model to different LLM choices?
- **RQ3:** How does the proposed IIDCL module influence performance and representation learning under different hyperparameter configurations?
- **RQ4:** How well does FedSCOPE handle cold-start scenarios where users have limited interaction histories?
- **RQ5:** Can federated training achieve better accuracy and generalization than standalone single-domain training?
- **RQ6:** Does the adaptive personalized differential privacy mechanism provide a better privacy–utility trade-off compared with conventional uniform DP?
- **RQ7:** Are the conclusions of FedSCOPE robust under varying federated configurations, such as the number of clients (*K*) and the per-round sampling ratio (*C*)?

Before presenting the evaluation results, we first introduce the datasets, baselines, evaluation metrics, and implementation details used in our experiments.

### Experimental settings

#### Datasets

We conduct experiments on the widely used Amazon e-commerce dataset^29^, which provides multi-domain user interaction histories and rich item metadata. This dataset is well suited for FedCDSR because it includes overlapping user behaviors across different domains. In our study, we focus on two domain pairs, namely Movie–Book and Food–Kitchen, which capture both complementary and heterogeneous user preferences, enabling a rigorous evaluation of cross-domain transfer. Following prior work^4,30^, we retain users who are active in both domains and exclude those with fewer than ten interactions. All interactions are chronologically ordered, with the last one used as the prediction target. The dataset is divided into training (80%), validation (10%), and test (10%) sets. Table 3 presents the statistics.

**Table 3:** Statistics of the experimental datasets.

Domain	#Users	#Items	#Train	#Test	#Valid
Movie	15,352	36,845	59,094	7,397	7,376
Book		63,937			
Food	16,579	29,207	40,557	5,080	5,059
Kitchen		34,886			

#### Baselines

We compare FedSCOPE with four categories of representative baselines: (1) traditional recommendation methods (ItemKNN^31^, BPRMF^32^); (2) cross-domain general recommendation methods (CoNet^13^, CCDR^33^); (3) cross-domain sequential recommendation methods ($\pi$-Net^14^, PSJNet^15^, MIFN^30^, C2DSR^4^, LEAGCN^16^, TJAPL^34^); and (4) federated cross-domain recommendation methods (FELLAS^8^, FedDCSR^7^, FedCSR^9^, FFMSR^35^). Following prior federated cross-domain recommendation studies (e.g., FedDCSR^7^, FedCSR^9^), methods not originally designed for federated settings are adapted using the standard FedAvg framework. This ensures a consistent and neutral federated optimization protocol across all baselines, so that performance differences primarily reflect model architectures rather than optimization discrepancies. All baselines follow the same data splits and evaluation protocol as FedSCOPE.

In this work, FedAvg is employed as a commonly adopted federated optimization framework to establish a controlled and reproducible experimental setting. Notably, FedSCOPE itself is not inherently dependent on any specific federated optimizer. The proposed semantic augmentation, decoupled intra–inter domain contrastive learning, and adaptive privacy mechanisms are designed at the model and objective levels, and thus remain applicable under other federated optimization schemes, such as FedProx^36^ or SCAFFOLD^37^. An extensive comparison across different federated frameworks is beyond the scope of this study and is left for future investigation.

#### Evaluation metrics

We adopt the widely used leave-one-out evaluation protocol^10,38^, where the last item in each user’s interaction sequence is held out as the test instance and ranked among 999 randomly sampled negative items^39^. We report results using three complementary metrics: $NDCG@\{5,10\}$, *MRR*, and $HR@\{5,10\}$. These metrics jointly assess ranking quality, recommendation precision, and hit accuracy at the top-*K* positions, where higher values indicate better recommendation performance.

#### Implementation details

All experiments are implemented in PyTorch and executed on machines equipped with NVIDIA GeForce RTX 4090 GPUs. For most baselines, hyperparameters are tuned via grid search on the validation set, and the optimal settings reported in their original papers are used when applicable. For our method, we set the embedding dimension to 64, mini-batch size to 256, and dropout rate to 0.3. The L2 regularization coefficient is searched from $\{10^{-5}, 5 \times 10^{-5}, 10^{-4}, 5 \times 10^{-4}\}$, and the learning rate from $\{10^{-4}, 5 \times 10^{-4}, 10^{-3}\}$. We train for 50 epochs with the Adam optimizer and apply early stopping if the validation loss does not improve for 10 epochs. The model with the highest validation *MRR* is selected for testing. Unless otherwise specified, we simulate $K=50$ clients in each scenario. In every communication round, a fraction $C=0.2$ of clients is sampled, each participating client performs $E=1$ local epoch, and the total number of rounds is $T=200$. All reported results are averaged over five runs with different random seeds, and we report the mean and standard deviation to ensure robustness and reproducibility.

### Experimental results and discussion

#### RQ1: Analysis of overall performance improvement

**Fig. 2:**
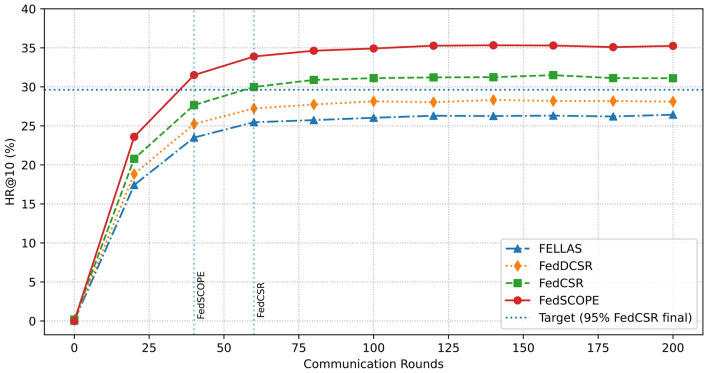
Convergence comparison in the kitchen-domain.

The overall performance comparison on the Movie–Book and Food–Kitchen datasets (Tables 4 and 5) reveals several important findings. First, cross-domain general recommendation methods (e.g., CoNet and CCDR) consistently outperform traditional methods (e.g., ItemKNN and BPRMF), highlighting the significance of domain adaptation and knowledge transfer in improving recommendation performance. Second, cross-domain sequential recommendation models further enhance accuracy by capturing temporal dependencies in user behavior, while attention-based approaches such as $\pi$-Net and TJAPL achieve even stronger results by modeling fine-grained preference dynamics.Among federated approaches, FedCSR exhibits competitive performance and demonstrates effective collaborative learning across distributed client data. Moreover, the recently proposed FFMSR^35^, which enhances cross-domain recommendation by jointly encoding raw textual semantics, integrating ID–Text modalities, and filtering irrelevant semantic noise, achieves better performance than FedCSR across all evaluated domains, demonstrating the benefit of incorporating richer semantic learning into federated CDR. While these baselines show competitive results, FedSCOPE attains the best or near-best performance on most metrics and datasets. Its performance gains are largely attributed to two design elements: (1) the decoupled intra- and inter-domain contrastive learning strategy, which promotes more stable cross-domain alignment, and (2) the privacy-preserving semantic enhancement module, which enriches user and item representations through LLM-generated semantic priors without exposing sensitive data. These components collectively enable FedSCOPE to deliver more robust and personalized recommendations in heterogeneous federated environments. Figure 2 further shows HR@10 over communication rounds. FedSCOPE demonstrates faster convergence than representative baselines (FedCSR, FedDCSR, and FELLAS), suggesting improved training efficiency and reduced communication overhead in federated settings.

**Table 4:** Experimental Results (%) on the Movie-Book Scenario.

Methods	Movie-domain Recommendation					Book-domain Recommendation				
	NDCG@5	NDCG@10	HR@5	HR@10	MRR	NDCG@5	NDCG@10	HR@5	HR@10	MRR
**Traditional Recommendation Methods**										
FedAvg+ItemKNN^31^	4.36	4.98	5.21	7.13	5.14	3.03	3.35	3.41	4.43	3.32
FedAvg+BPRMF^32^	4.78	5.12	5.51	7.43	5.63	3.46	3.41	3.46	4.38	3.45
**Cross-domain General Recommendation Methods**										
FedAvg+CoNet^13^	7.52	8.22	8.39	10.56	8.31	6.32	6.52	6.65	7.44	6.69
FedAvg+CCDR^33^	7.68	8.27	8.74	10.61	8.33	6.37	6.57	6.79	7.49	6.75
**Cross-domain Sequential Recommendation Methods**										
FedAvg+$\pi$-Net^14^	11.46	11.91	12.49	11.85	8.93	9.58	9.77	9.99	10.58	9.91
FedAvg+PSJNet^15^	11.88	12.57	13.04	15.27	12.37	9.81	10.09	10.32	11.02	10.18
FedAvg+MIFN^30^	11.95	12.94	13.25	16.03	12.79	9.86	10.05	10.25	10.81	10.25
FedAvg+C2DSR^4^	12.56	13.51	14.21	17.29	13.28	9.91	10.19	10.58	11.49	10.29
FedAvg+LEAGCN^16^	12.97	13.91	15.22	18.03	13.86	11.21	11.36	11.01	11.99	10.82
FedAvg+TJAPL^34^	14.28	14.99	16.35	19.75	14.97	12.63	12.76	12.49	13.43	11.87
**Federated Cross-domain Recommendation Methods**										
FELLAS^8^	22.79	23.23	23.82	27.71	23.24	18.53	19.32	20.37	22.52	21.37
FedDCSR^7^	23.18	23.62	24.21	28.09	24.63	18.92	20.71	21.76	23.91	22.76
FedCSR^9^	26.13	26.57	27.16	31.04	26.58	21.87	22.66	23.71	25.86	24.71
FFMSR^35^	26.62	27.34	27.95	32.02	27.01	22.31	23.08	24.12	25.96	25.09
**FedSCOPE (Ours)**	**29.07**	**31.64**	**32.32**	**36.11**	**31.95**	**25.97**	**27.55**	**28.41**	**31.15**	**29.06**

**Table 5:** Experimental Results (%) on the food-kitchen scenario.

Methods	Food-domain Recommendation					Kitchen-domain Recommendation				
	NDCG@5	NDCG@10	HR@5	HR@10	MRR	NDCG@5	NDCG@10	HR@5	HR@10	MRR
**Traditional Recommendation Methods**										
FedAvg+ItemKNN^31^	5.47	5.78	6.32	8.06	6.05	3.41	3.58	4.12	5.39	4.02
FedAvg+BPRMF^32^	5.68	6.16	6.94	8.54	6.23	3.58	3.98	4.31	5.56	4.24
**Cross-domain General Recommendation Methods**										
FedAvg+CoNet^13^	8.85	9.38	10.01	11.59	9.37	6.74	7.35	7.31	8.95	7.41
FedAvg+CCDR^33^	9.18	9.75	10.34	12.14	9.73	6.81	7.27	7.47	9.34	7.65
**Cross-domain Sequential Recommendation Methods**										
FedAvg+$\pi$-Net^14^	15.06	15.87	16.99	19.49	15.42	10.72	11.47	12.08	14.41	11.27
FedAvg+PSJNet^15^	15.81	16.51	18.02	20.19	16.07	11.42	12.06	12.91	14.89	11.84
FedAvg+MIFN^30^	16.02	16.75	18.17	20.45	16.29	11.31	12.03	12.60	14.82	11.83
FedAvg+C2DSR^4^	16.39	17.45	18.98	22.28	16.65	11.98	12.68	13.48	15.92	12.39
FedAvg+LEAGCN^16^	17.23	18.22	19.73	23.12	17.86	13.42	13.81	14.72	17.24	13.46
FedAvg+TJAPL^34^	17.98	18.97	20.42	23.75	18.69	13.86	14.52	15.18	18.33	14.59
**Federated Cross-domain Recommendation Methods**										
FELLAS^8^	20.94	21.53	21.69	26.12	21.04	16.53	16.88	17.81	20.04	15.72
FedDCSR^7^	22.36	22.95	23.11	27.54	22.46	17.95	18.34	19.23	21.46	19.14
FedCSR^9^	25.42	26.01	26.17	30.63	23.52	21.01	21.36	22.43	24.52	22.29
FFMSR^35^	25.73	26.26	26.34	31.01	24.03	21.68	22.11	23.36	25.18	22.85
**FedSCOPE (Ours)**	**30.56**	**31.62**	**32.43**	**34.64**	**27.56**	**27.49**	**28.58**	**29.02**	**30.13**	**26.74**

#### RQ2: Contribution and sensitivity of LLM-based enhancement

**Fig. 3:**
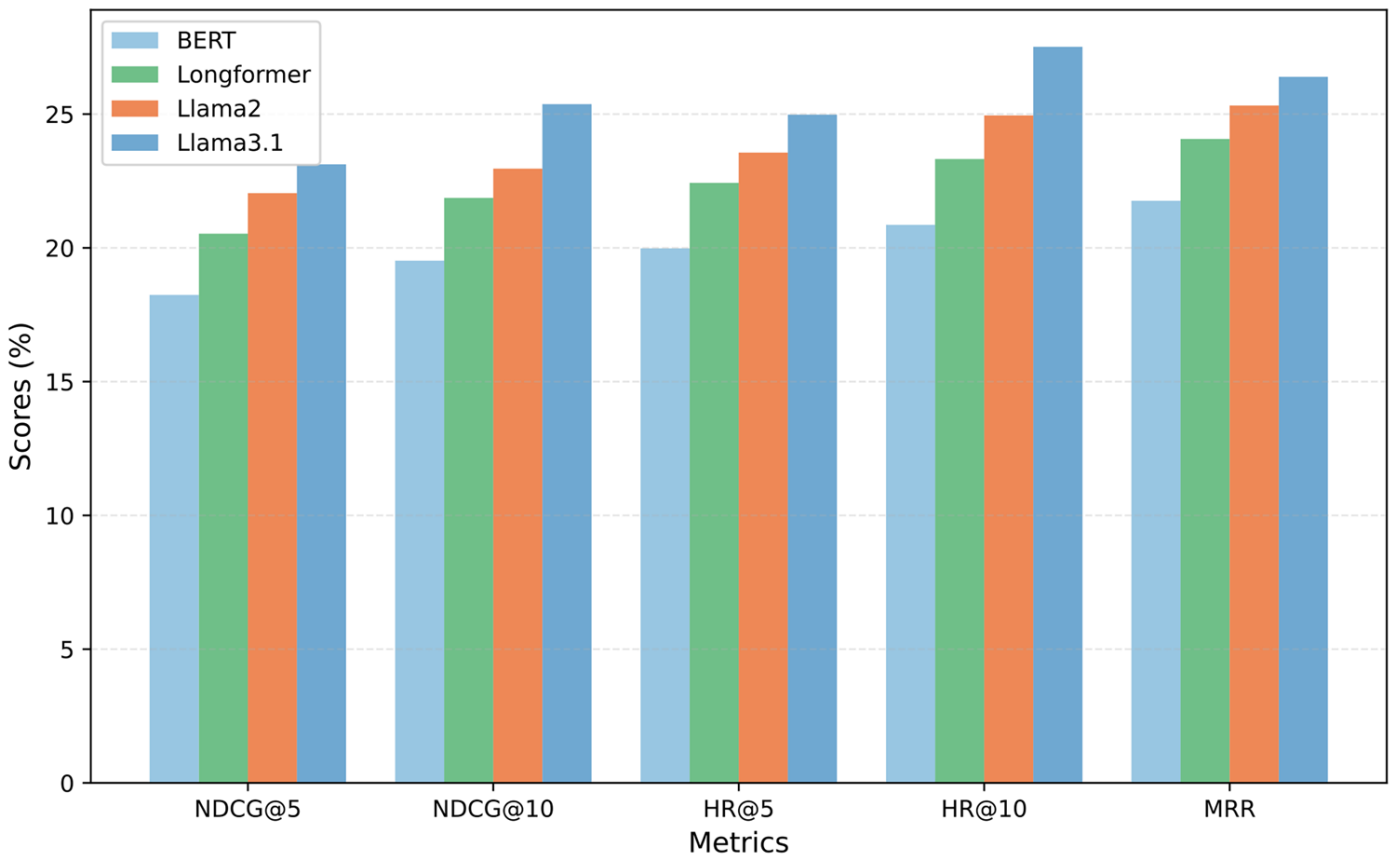
Performance comparison across different models in the Book-domain.

To evaluate the contribution of LLM-based semantic augmentation, we conducted ablation experiments by selectively removing the user feature enhancement (UFE) and item attribute enhancement (IAE) modules. As shown in Table 6, removing both modules leads to the lowest performance, confirming the necessity of LLM-based augmentation. Retaining only UFE yields slightly better results than retaining only IAE, indicating that user-side enrichment plays a more critical role in modeling behavioral sequences, likely because user-level representations directly shape sequential preference modeling. Nevertheless, IAE also contributes by enriching item-level semantics, and combining UFE and IAE achieves the best results in both domains, demonstrating their complementary nature. We further analyzed the sensitivity of FedSCOPE to different LLMs by comparing BERT, Longformer, Llama2, and Llama3.1 on the Book domain. As shown in Fig. 3, FedSCOPE consistently outperforms the baseline FedSeqRec across all tested LLMs, highlighting its robustness in leveraging diverse semantic representations. BERT shows relatively weaker performance due to its limited capacity for long-context modeling, while Longformer better captures extended user sequences. More advanced models, such as Llama2 and Llama3.1, bring larger improvements, suggesting that model scale and the richness of pretraining data are positively correlated with recommendation quality. Importantly, these results demonstrate that FedSCOPE is not tied to a specific LLM and can effectively benefit from a wide range of language models. Beyond accuracy, we also examined the trade-off between computational overhead and performance gains. BERT offers the lowest computational cost and memory footprint, though its improvements are modest. Longformer requires moderately higher resources due to its extended attention mechanism but achieves noticeably better performance on long sequences. Llama2 and Llama3.1 demand the highest offline computational resources, yet deliver the most significant accuracy gains, especially in sparse and heterogeneous domains. Since FedSCOPE performs augmentation entirely offline and caches the generated features, these costs are incurred only once, avoiding repeated overhead during federated training and keeping the additional communication burden negligible. This design enables flexible choices: lightweight models are preferable in resource-limited scenarios, whereas larger models are more suitable when maximizing accuracy is the priority, making FedSCOPE adaptable to a wide range of real-world deployments.

**Table 6:** Performance comparison (%) with user feature and item attribute enhancement.

Component		Food-domain			Kitchen-domain		
UFE	IAE	NDCG@10	HR@10	MRR	NDCG@10	HR@10	MRR
$\times$	$\times$	12.45	13.05	9.10	10.73	11.08	10.15
$\times$	✓	20.69	20.27	17.86	18.43	20.98	19.86
✓	$\times$	21.43	20.96	18.57	19.21	22.16	21.95
✓	✓	**31.59**	**34.58**	**27.49**	**29.12**	**31.47**	**27.32**

#### RQ3: Effectiveness and sensitivity analysis of IIDCL

To evaluate the effectiveness of the proposed IIDCL mechanism and analyze its key hyperparameters, we conduct a comprehensive sensitivity study on $\lambda _{\text {intra}}$, $\lambda _{\text {inter}}$, and the overall contrastive weight $\alpha$ in both the Book-Movie and Food-Kitchen scenarios.

**Fig. 4:**
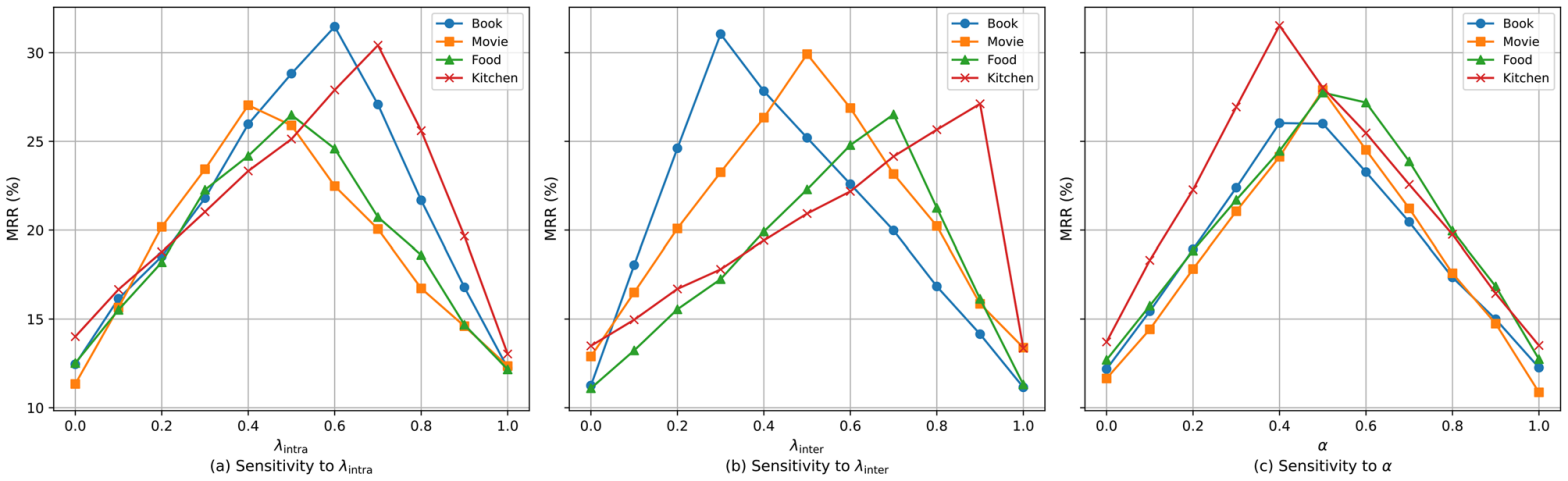
Sensitivity analysis of key hyperparameters in IIDCL. (a) Impact of $\lambda _{\text {intra}}$. (b) Impact of $\lambda _{\text {inter}}$. (c) Impact of the overall contrastive weight $\alpha$.

**Sensitivity of **$\lambda _{\text {intra}}$. We first analyze the impact of the intra-domain contrastive weight $\lambda _{\text {intra}}$ by varying it from 0 to 1.0 with a step size of 0.1, while fixing $\lambda _{\text {inter}}$ and $\alpha$ to their default values. As shown in Fig. 4, increasing $\lambda _{\text {intra}}$ initially leads to consistent performance improvements, indicating that stronger intra-domain contrastive regularization effectively enhances user aggregation and personalization within each domain. However, when $\lambda _{\text {intra}}$ becomes excessively large, performance starts to degrade, suggesting that overemphasizing intra-domain consistency may suppress cross-domain knowledge transfer and reduce generalization capability.

**Sensitivity of **$\lambda _{\text {inter}}$. Next, we examine the sensitivity of the inter-domain contrastive weight $\lambda _{\text {inter}}$ by varying it within the same range while fixing $\lambda _{\text {intra}}$ and $\alpha$. Moderate values of $\lambda _{\text {inter}}$ significantly improve cross-domain separability by encouraging representations of the same user across different domains to align while pushing apart different users. Nevertheless, overly large $\lambda _{\text {inter}}$ weakens domain-specific personalization, leading to performance degradation. These results confirm that excessive emphasis on inter-domain alignment may override fine-grained domain-level preferences.

**Sensitivity of **$\alpha$. Finally, we investigate the influence of the global contrastive weight $\alpha$, which balances the prediction loss and the contrastive objectives. We observe that FedSCOPE achieves stable performance across a broad range of $\alpha$ values, indicating that IIDCL provides effective regularization without requiring precise tuning of the overall contrastive strength. Extremely small $\alpha$ values underutilize contrastive signals, while overly large values may dominate the prediction objective, slightly degrading accuracy. The sensitivity analysis demonstrates that $\lambda _{\text {intra}}$, $\lambda _{\text {inter}}$, and $\alpha$ play complementary roles in IIDCL. Optimal performance emerges when these hyperparameters are properly balanced, validating the design motivation of decoupling intra-domain personalization and inter-domain generalization within the proposed framework.

**Fig. 5:**
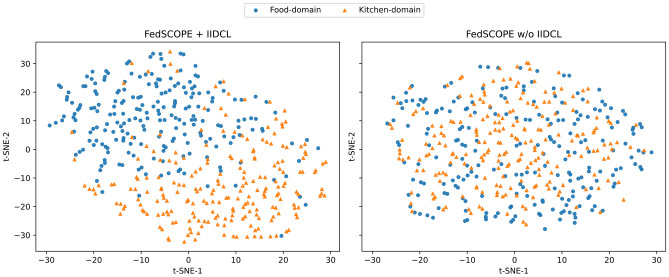
t-SNE visualization of user representations with and without IIDCL.

**Visualization and analysis.** Beyond quantitative results, we further visualize user representations using t-SNE to provide intuitive insights into the role of IIDCL. As illustrated in Fig. 5, removing IIDCL results in scattered intra-domain representations with substantial inter-domain overlap, reflecting weak within-domain consistency and limited cross-domain discriminability. In contrast, FedSCOPE with IIDCL learns more compact intra-domain clusters and clearer inter-domain separation, where positive samples are pulled closer and negative samples are pushed farther apart. Such structured representations enable the model to simultaneously capture domain-specific behavioral patterns and preserve transferable knowledge across domains.

#### RQ4: Performance under cold-start scenarios

Table 7 reports the performance of different methods under cold-start scenarios, where users exhibit limited historical interactions. Compared with FedCSR, FedSCOPE consistently achieves better performance across all four domains, suggesting an improved ability to cope with data sparsity in federated cross-domain recommendation settings. When the LLM-based semantic augmentation module is removed, the performance generally decreases across domains, indicating that relying solely on behavioral signals may be less effective for representing cold-start users. This performance gap tends to be more noticeable in domains with sparser user interactions, highlighting the potential benefit of incorporating semantic information to complement limited behavioral data. By integrating LLM-generated user and item semantics, FedSCOPE can leverage additional contextual cues beyond short interaction histories, which helps mitigate the cold-start challenge to some extent.

**Table 7:** Performance comparison under user cold-start scenarios (users with $\le 5$ interactions, %).

Method	Book-domain		Movie-domain		Food-domain		Kitchen-domain	
	**HR@10**	**NDCG@10**	**HR@10**	**NDCG@10**	**HR@10**	**NDCG@10**	**HR@10**	**NDCG@10**
FedCSR	17.84	15.62	18.31	16.05	15.26	13.48	14.92	13.21
FedSCOPE w/o LLM	22.97	20.84	23.41	21.36	19.73	17.92	18.66	16.85
**FedSCOPE**	**27.96**	**25.41**	**28.87**	**26.02**	**24.18**	**22.03**	**23.07**	**21.14**

#### RQ5: Comparison of federated training and standalone training

**Fig. 6:**
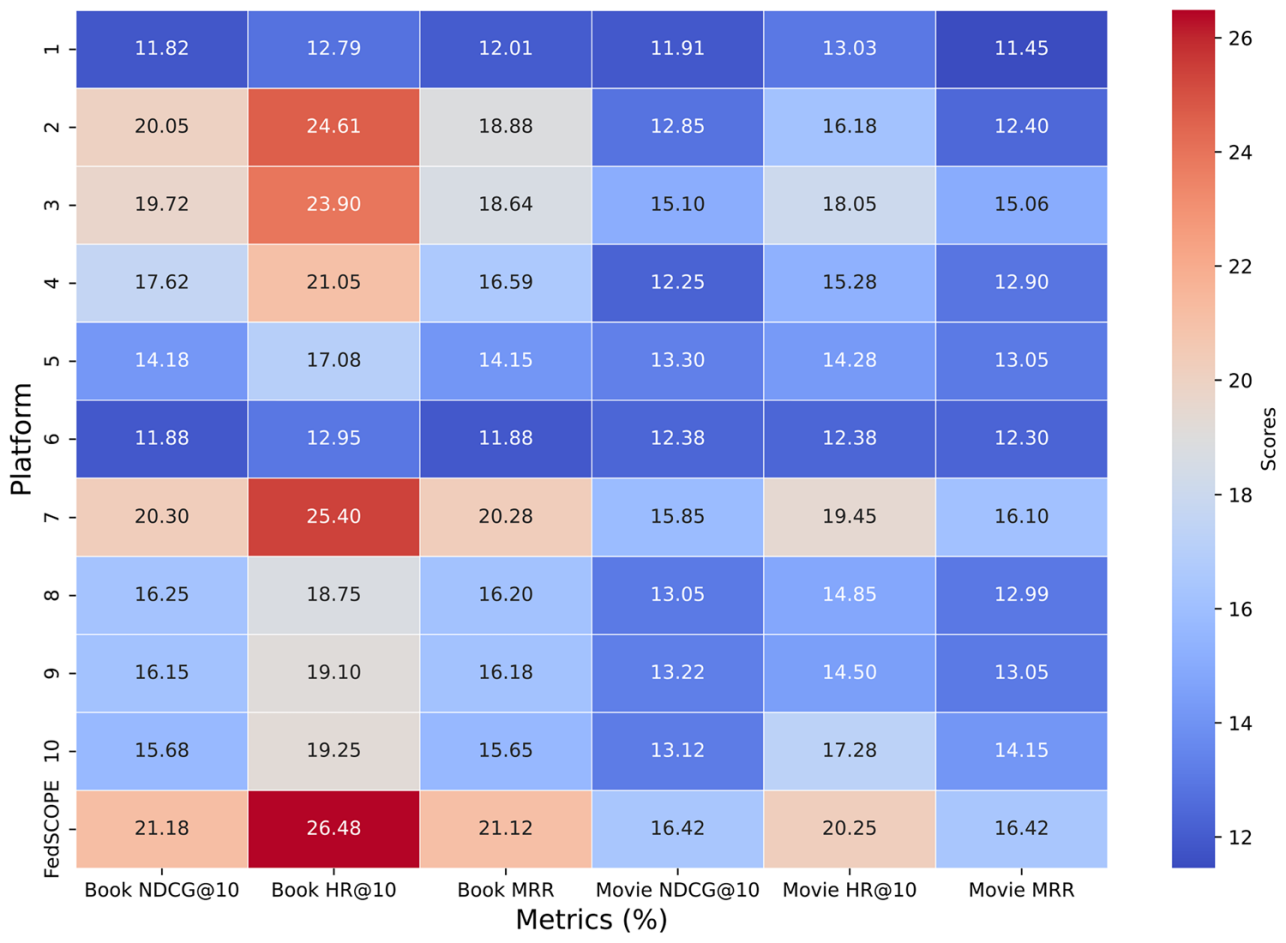
Comparison of recommendation performance between FedSCOPE and independent training in the Book-Movie scenario.

To further evaluate the effectiveness of cross-platform collaboration, we compared FedSCOPE with independently trained models in the Movie–Book scenario. Independent training refers to a setting where each platform trains its own CSR model locally without any information exchange, whereas FedSCOPE collaboratively constructs a unified federated CSR model while keeping data decentralized. As shown in Fig. 6, FedSCOPE consistently outperforms independently trained models across all platforms, demonstrating the advantages of collaborative knowledge integration. This joint optimization allows FedSCOPE to capture complementary user behavior patterns and cross-domain correlations that isolated models fail to exploit. The results also highlight the strong impact of data sparsity on model performance. In independent training, platforms with larger amounts of data achieve relatively better results, while those with limited data suffer from lower accuracy due to insufficient behavioral signals. In contrast, FedSCOPE effectively mitigates these limitations by aggregating knowledge across platforms, enriching representation learning, and significantly improving overall recommendation performance even in sparse data conditions.

#### RQ6: Privacy–utility trade-off of adaptive DP

**Fig. 7:**
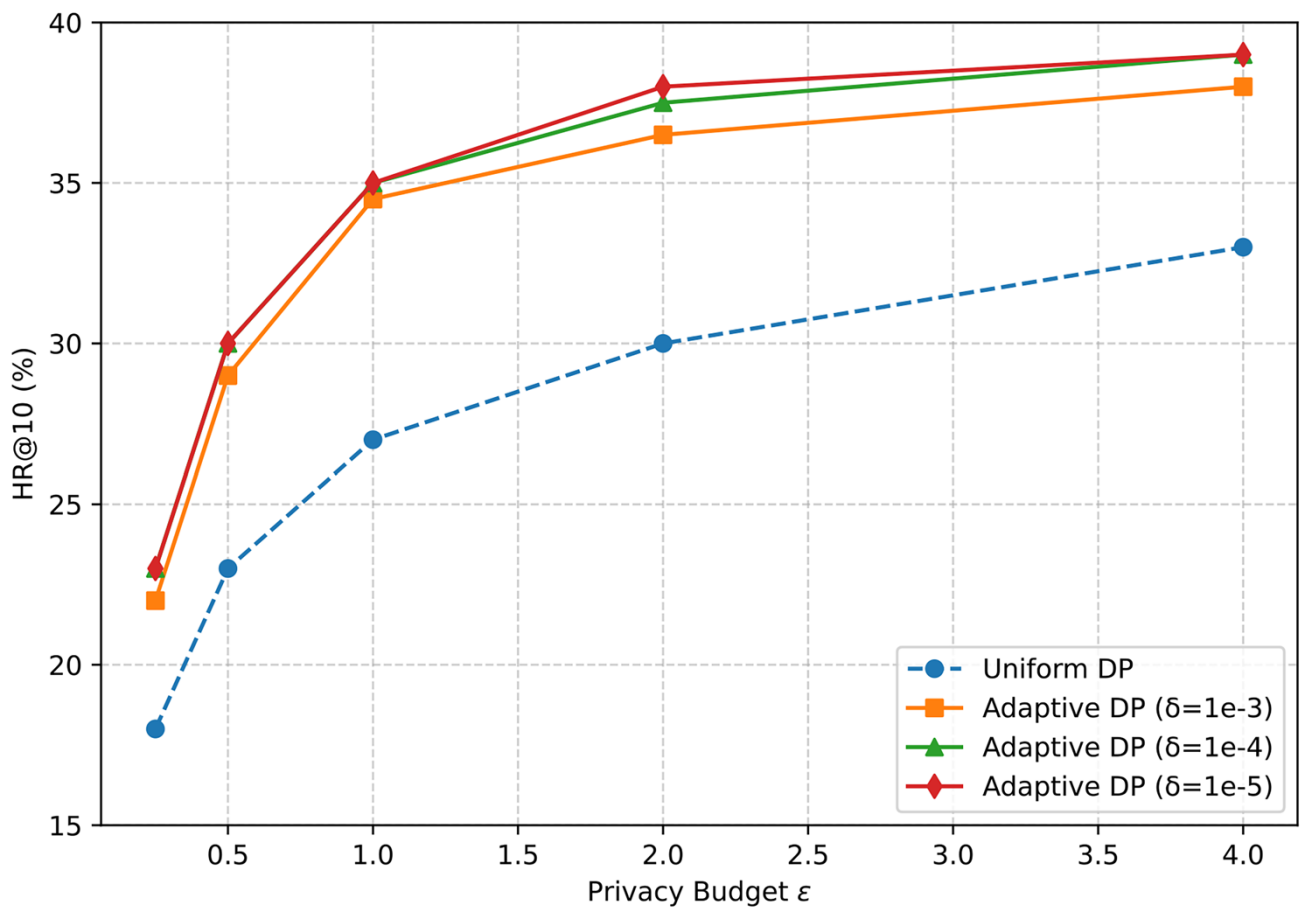
Kitchen domain: Adaptive vs. Uniform DP under different $\delta$.

We next examine how Adaptive DP influences the privacy–utility trade-off compared with a standard Uniform DP mechanism. We evaluate both approaches under identical training protocols in the Kitchen domain across a range of privacy budgets $\epsilon \in \{0.25, 0.5, 1, 2, 4\}$. As shown in Fig. 7, recommendation performance (HR@10) increases monotonically with larger $\epsilon$ for all methods, reflecting the expected reduction in injected noise. Importantly, every Adaptive DP variant consistently lies above the Uniform DP baseline across all privacy levels. The relative advantage is most pronounced under stricter privacy (smaller $\epsilon$) and gradually narrows as the constraint relaxes. This behavior can be attributed to two design factors: (i) personalized budget allocation, which avoids over-noising clients with abundant data while preventing under-protection for those with limited data; and (ii) adaptive clipping, which stabilizes per-client sensitivity and reduces the effective noise required by the Gaussian mechanism. Together, these mechanisms enable more efficient noise allocation while maintaining strong privacy guarantees. To further assess robustness, we vary the failure probability parameter $\delta \in \{10^{-3}, 10^{-4}, 10^{-5}\}$. The three Adaptive DP curves in Fig. 7 almost overlap, indicating that performance is largely insensitive to $\delta$. This aligns with theoretical expectations, since $\delta$ controls only a negligible failure probability rather than the dominant privacy–utility balance. This robustness also simplifies hyperparameter selection in practical deployments.

#### RQ7: Robustness to the federated configuration

**Fig. 8:**
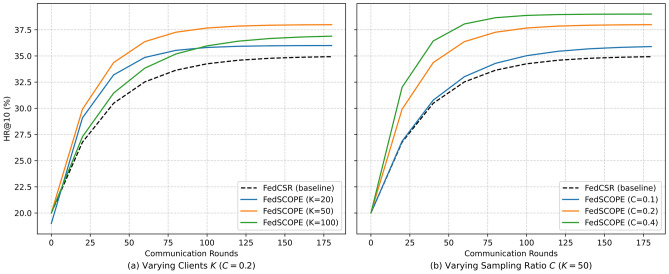
Robustness to the federated configuration in the Kitchen domain. (**a**) Varying the number of clients *K* with $C{=}0.2$. (**b**) Varying the sampling ratio *C* with $K{=}50$.

We further evaluate the robustness and scalability of FedSCOPE under different federated configurations. Specifically, we vary the number of clients $K \in \{20, 50, 100\}$ with a fixed sampling ratio $C = 0.2$, and vary the per-round sampling ratio $C \in \{0.1, 0.2, 0.4\}$ with a fixed number of clients $K = 50$. All experiments use the same dataset, training protocol, and privacy parameters. As shown in Fig. 8, increasing *K* from 20 to 100 (panel a) leads to slightly slower convergence due to stronger client heterogeneity and reduced per-client data volume, resulting in a minor drop in the final HR@10. However, the performance degradation remains small, and the relative improvements of FedSCOPE over baseline methods are consistently preserved. When increasing *C* from 0.1 to 0.4 (panel b), convergence becomes noticeably faster and the final accuracy improves slightly, since more clients participate in each round. Although a larger *C* accelerates per-round privacy consumption in the privacy accountant, the overall privacy–utility balance remains stable under the same global budget. This trade-off allows practitioners to adjust *C* flexibly based on system constraints and application requirements.

## Conclusion

In this work, we proposed FedSCOPE, a federated cross-domain sequential recommendation framework that addresses three practical challenges: data sparsity, domain heterogeneity, and the privacy–utility trade-off. FedSCOPE integrates three complementary components: an offline LLM-based semantic augmentation module to enrich sparse behavioral representations, an Intra- and Inter-Domain Decoupled Contrastive Learning mechanism to enhance both personalization and cross-domain alignment, and an adaptive personalized differential privacy strategy that tailors noise injection to client-specific data characteristics. Through joint optimization within a secure federated protocol, FedSCOPE achieves consistently superior performance on multiple real-world datasets, delivering higher accuracy, stronger generalization, and a more favorable privacy–utility balance, together with faster convergence. Future work will explore more advanced semantic enrichment, communication-efficient optimization, and adaptive privacy mechanisms to further improve scalability and robustness in federated cross-domain recommendation.

## Data Availability

The datasets generated and analysed during the current study are available from the corresponding author on reasonable request.
